# Supplementary material for: Leaf Extracts of Invasive Woody Species Demonstrate Allelopathic Effects on the Growth of a Lawn Grass Mixture
Source: Plants (Basel). 2023 Dec 6;12(24):4084. doi: 10.3390/plants12244084 (PMC10747084; doi:10.3390/plants12244084)
Supplement: Supplementary file 1 [file plants-12-04084-s001.zip › plants-2747564-supplementary.pdf]

Table S1. Profiles of water extracts of leaf litter, mass. % of the extract.

	Water extracts of leaf litter of				
	Acer negundo	*Ailanthus altissima*	*Elaeagnus angustifolia*	*Quercus rubra*	*Robinia pseudoacacia*
	Phenolic and aliphatic acids				
Acetic acid	6.53±0.66[a]	0.11±0.03[c]	0.77±0.09[b]	t	0.17±0.5[c]
Butanedioic acid	8.60±1.12[a]	4.22±0.91[c]	5.68±0.84[b]	6.70±1.05[b]	2.94±0.51[d]
Tartaric acid	1.02±0.33[a]	0.66±0.12[b]	t	1.51±0.19[a]	0.31±0.08[c]
Malic acid	0.59±0.12[c]	3.59±0.44[a]	t	2.30±0.24[b]	0.53±0.18[c]
Vanillic acid	2.09±0.31[b]	t	3.80±0.42[a]	t	0.37±0.11[c]
Protocatechuic acid	t	t	0.99±0.18[b]	15.45±2.07[a]	t
Quininic acid	11.19±1.28[a]	2.10±0.42[b]	0.12±0.09[c]	t	0.20±0.09
Idonic acid	10.28±1.57[a]	0.12±0.04[c]	t	0.16±0.04[c]	0.66±0.11[b]
Glucuronic acid	0.35±0.08[b]	1.87±0.25[a]	0.47±0.17[b]	0.44±0.09[b]	0.18±0.04[c]
Glucopyranuronic acid	t	1.05±0.11[b]	t	21.34±2.18[a]	t
Pentanoic acid	t	t	11.84±1.55[a]	t	0.30±0.07[b]
Total content % (number of compounds)	41.3 ±2.09[b] (11)	19.20±1.50 [d] (25)	35.78±1.35 [c] (13)	62.9 ±1.64[a] (14)	19.23±1.23[d] (44)
	Carboxylic esters				
Hexanedioic acid Dimethyl ester	t	0.09±0.04[b]	t	t	20.69±2.27[a]
Total content % (number of compounds)	0.93[d] (1)	1.24[c] (2)	3.32[b] (1)	<0.01%	20.87[a] (2)
	Carbohydrates and their derivatives				
D-erythro-2-pentulose	4.25±1.07[b]	0.17±0.07[c]	9.71±1.33[a]	3.75±0.86[b]	t
Methyl-a-D-glucofuranoside	6.50±1.24[a]	0.95±0.17[d]	0.55±0.13[e]	3.68±0.49[b]	1.73±0.33[c]
Glyceryl-glycoside	t	3.43±0.71[a]	t	t	0.41±0.14[b]
D-Psicofuranose	0.12±0.05[c]	7.45±0.81[a]	1.50±0.33[b]	1.70±0.29[b]	0.21±0.08[c]
D-Allofuranose	0.42±0.12[b]	0.31±0.09[b]	t	0.18±0.05[c]	4.96±0.55[a]
D-(+)-Glucosamine	t	0.18±0.05[b]	t	6.86±0.95[a]	t
L-(-)-Sorbose	0.21±0.09[b]	t	t	t	7.96±1.15[a]
Total content % (number of compounds)	15.18±0.81d (10)	27.99 ±1.10[b] (17)	16.11± 0.87[d] (9)	20.10±1.25[c] (9)	40.14±0.87[a] (48)
	Polyphenols				
L-(-)-Arabitol	1.70±0.38[b]	0.73±0.22[c]	4.35±0.49[a]	0.32±0.11[d]	0.05±0.02[e]
D-Fucitol	1.14±0.25[b]	0.02±0.01[d]	1.98±0.56[a]	2.41±0.63[a]	0.13±0.03[c]
1,5-Angidroglucitol	4.35±0.75[a]	0.03±0.01[d]	2.41±0.55[b]	3.21±0.59[a]	0.38±0.11[c]
Glycerol	7.21±1.15[a]	4.01±1.01[b]	8.53±1.55[a]	0.29±0.07[c]	7.59±1.50[a]
Erythritol / Meso-Erythritol	7.37±0.95[a]	0.24±0.11[e]	3.85±0.35[b]	2.87±0.40[c]	1.22±0.42[d]
1,2,2-3-Butanetriol	t	t	t	3.98±0.78[a]	0.07±0.02[b]
Dulcitol	t	3.21±0.65[a]	0.07±0.02[b]	t	t

Adonitol	t	18.06±2.15^{a}	t	t	0.12±0.04^{b}
Total content % (number of compounds)	22.81±0.65^{c} (8)	30.73±0.73^{a} (16)	27.53±0.74^{b} (9)	16.31±1.05^{d} (8)	15.42±0.85^{d} (22)
	Glycosides				
L-(+)-Ramnopyranose	6.25±0.81^{a}	t	t	t	0.57±0.12^{b}
Arbutoside	0.64±0.12^{a}		0.25±0.07^{b}	0.09±0.02^{c}	0.07±0.02^{c}
Arbutin	t	13.80±1.98^{a}	t	t	0.21±0.03^{b}
Total content % (number of compounds)	6.91±0.51^{b} (3)	15.07±0.68^{a} (3)	0.37 ±0.05^{d} (2)	0.11±0.03^{e} (2)	0.95±0.12^{c} (4)
	Nitrogen Compounds				
Meglumine	4.78±0.85^{a}	t	t	t	0.14±0.05^{b}
L-Valine	2.20±0.55^{a}	t	t	0.19±0.06^{b}	0.12±0.03^{b}
L-Ornitine	1.97±0.22^{b}	0.10±0.02^{c}	4.76±1.17^{a}	0.17±0.05^{c}	0.04±0.01^{d}
Asparagine	t	t	4.84±0.92^{a}	t	0.04±0.01^{b}
Total content % (number of compounds)	9.59±0.35^{b} (5)	0.63±0.05^{c} (6)	12.83±0.58^{a} (5)	0.36±0.04^{e} (2)	2.60±0.38^{d} (6)

Mean the five determinations ± standard deviation. ; t –compound content less than 0.01%; different letters indicate significant differences among five layers using post-hoc test ($p<0.05$).

Table S2. Elemental composition of leaf litter, mass % in ashes.

	Acer negundo	*Ailanthus altissima*	*Elaeagnus angustifolia*	*Quercus rubra*	*Robinia pseudoacacia*
O	41.39±1.92^{a}	39.98±1.83^{a}	38.58±1.24^{a}	38.58±0.91^{a}	39.67±1.56^{a}
C	24.94±1.23^{a}	26.98±1.17^{a}	25.78±1.99^{a}	25.78±1.89^{a}	18.06±1.27^{b}
Ca	18.46±1.83^{a}	10.42±1.06^{c}	15.49±1.39^{b}	12.96±0.8^{c}	11.91±0.82^{c}
K	7.69±0.33^{c}	9.53±0.40^{b}	12.88±1.22^{a}	4.22±0.87^{d}	14.26±1.49^{a}
Mg	4.83±0.24^{b}	3.82±0.17^{c}	2.71±0.19^{d}	4.51±0.35^{b}	8.33±0.54^{a}
P	0.75±0.08^{c}	4.46±0.13^{a}	0.99±0.11^{c}	0.88±0.17^{c}	1.90±0.19^{b}
Si	1.44±0.12^{a}	1.37±0.12^{a}	0.55±0.07^{c}	1.55±0.18^{a}	0.87±0.08^{b}
Mn	1.28±0.16^{c}	2.33±0.10^{a}	0.71±0.12^{d}	0.54±0.12^{d}	1.78±0.21^{b}
Mo	1.01±0.19^{c}	2.00±0.17^{a}	1.35±0.29^{b}	0.76±0.23^{d}	1.57±0.22^{b}
S	0.37±0.11^{d}	0.91±0.1b^{c}	1.67±0.20^{a}	0.72±0.03^{c}	0.92±0.09^{b}
Al	0.20±0.03^{c}	0.53±0.04^{a}	0.16±0.02^{c}	0.36±0.04^{b}	0.14±0.01^{c}
Zn	0.02±0.01^{c}	0.41±0.01^{b}	1.09±0.09^{a}	0.39±0.10^{b}	0.47±0.12^{b}

Mean the five determinations ± standard deviation. ; different letters indicate significant differences among five layers using post-hoc test ($p<0.05$).
